# A Novel Method for γ-Aminobutyric Acid Biosynthesis Using Glutamate Decarboxylase Entrapped in Polyvinyl Alcohol–Sodium Alginate Capsules

**DOI:** 10.3390/molecules28196844

**Published:** 2023-09-28

**Authors:** Fei Zhu, Sheng Hu, Weirui Zhao, Lehe Mei

**Affiliations:** 1 Department of Food Science, Zhejiang Pharmaceutical University, Ningbo 315000, China; 2 School of Biological and Chemical Engineering, NingboTech University, Ningbo 315000, China; 3 College of Chemical and Biochemical Engineering, Zhejiang University, Hangzhou 310058, China; 4 Jinhua Advanced Research Institute, Jinhua 321019, China

**Keywords:** glutamate decarboxylase, γ-aminobutyric acid, immobilization, polyvinyl alcohol, sodium alginate, capsules, biosynthesis

## Abstract

γ-aminobutyric acid (GABA) has essential physiological functions in the human body. A novel method using glutamate decarboxylase (GAD) entrapped in polyvinyl alcohol (PVA)-sodium alginate (SA) capsules provides a green biological strategy for GABA synthesis. In this investigation, the stability range of immobilized GAD was effectively broadened, and immobilized GAD could be repeatedly used as a batch and fixed-bed column catalyst. The immobilized enzymes were stable and retained 89% of their activity in a pH range of 4.0–5.6, while there was an approximately 50% decrease in free GAD activity in the pH range of 4.8 ± 0.4. The immobilized GAD affinity to the substrate improved, and this was evidenced by the apparent decrease in *K*_m_ to 13.3 mmol/L from the 30.9 mmol/L for free GAD. The immobilized GAD retained >90.6% activity after eight cycles and a near-100% enzyme activity retention after 120 h of a continuous fixed-bed column catalyst operation. This study has thus presented an effective PVA–SA–GAD immobilization method that could be used to continuously scale-up GABA biosynthesis.

## 1. Introduction

γ-aminobutyric acid (GABA) is a neurotransmitter inhibitor in the mammalian nervous system, which can reportedly inhibit excessive excitation and eliminate neurogenic tonus in the central nervous system [1,2]. GABA has been identified in several practical applications in the pharmaceutical industry in relation to human health, the food industry, and agriculture. GABA can reportedly increase the level of human growth hormone and improve mental condition and sleep quality, and consequently, the daily intake of GABA-containing foods is expected to help control blood pressure [3,4,5]. GABA has also been recognized as playing an important role in enhancing immunity, fighting obesity, and improving visual cortical function [6,7,8]. Furthermore, GABA was found to increase drought stress tolerance in plants and increase early growth and photosynthesis in maize [9,10]. 

GABA can be synthesized by potassium phthalimide reactions with 4-chloro-butyronitril, or by methyl bromoacetate reacting with ethylene under several reaction processes. However, the direct addition of chemical GABA to food is considered unnatural and unsafe [11,12]. Consequently, there is great interest in the development of natural methods by which to produce GABA. Edible plants and natural microorganisms isolated from foods are good sources of natural GABA. Research has shown that GABA can been separated from several plant sources, such as tea, mulberry leaves, tomato, and fermented soybean [13,14,15,16]. GABA can also be obtained by natural microorganism fermentation, such as with fungi, bacteria, and yeast. For example, *Rhizopus oligosporus,* when anaerobically incubated with steamed soybeans [16], *Corynebacterium glutamicum* [17], and yeast when fermented in soybean residue [18] have all been found to produce GABA. Lactic acid bacteria (LAB) fermentation, however, is considered one of the safest methods by which to produce GABA. There is a diverse range of GABA producing species in the LAB, including *Lactobacillus brevis* [19,20], *Lactobacillus delbrueckii* subsp. bulgaricus [21,22], *Lactobacillus fermentum* [23,24], and *Lactobacillus plantarum* [25,26].

Microorganisms have a variety of different bacterial catalysis strategies for GABA production, but their applications are currently limited. Bacterial catalysis requires highly sterile operating conditions throughout the process, and the downstream steps for product separation from the fermentation broth require additional economic inputs, which increases the final production cost. Glutamate decarboxylase (GAD) from LAB is a natural decarboxylase which can catalyze L-sodium glutamate to transform into GABA in microorganisms. It can also trigger reactions to produce GABA with pyridoxal-5-phosphate in vitro. The single substrate “one step” reaction makes it extremely easy to separate GABA from the reaction system. However, the applications of the free enzymes used for GABA production are simultaneously limited due to the activity loss and low stability and recovery rates caused by the usage of disposable enzymes [27]. Immobilization of the enzyme with supports is an efficient method by which to improve stability and reusability of the enzyme as well as product separability [28,29]. Compared with the free GAD catalytic process for GABA production, the immobilization of GAD could reduce total enzyme activity loss by improving enzyme stability and the inclusion of repeat cycling for the enzymatic process.

Proper immobilization support is essential for enzymes. The immobilization performance of an enzyme is closely related to the materials and structure of the support. If the reaction medium contains solids or shares physico-chemical similarities with immobilization supports, the reuse of the biocatalyst requires additional processing beyond filtration [30]. The strategy of immobilization must consider potential decreases in enzyme stability resulting from enzyme structure change after interaction with the support [31,32]. A successful immobilization method will solve the problem of inhibition from the product accumulation in the internal microenvironment [33]. It is also necessary to consider inversion of enzyme specificity after immobilization, which has also been reported in the cases of certain supports [34,35].

Polyvinyl alcohol (PVA) which has been utilized in the biological and medical field, is a low cost, safe, and friendly material for use with enzymes and microorganisms that has a high mechanical strength, chemical inertness, and good water retention [36,37,38]. It is consequently a potential superior immobilization support for GAD. Sodium alginate (SA) as a subsidiary beading agent could be combined and used with PVA to enhance the mechanical strength of the gels in the cell or during enzyme immobilization. The hydrogel formed by PVA or PVA–SA has reportedly been applied in wastewater treatments, such as for phosphorous removal, dye absorption, and arsenic elimination [39,40,41]. Furthermore, some studies have found that PVA can be utilized for cell [42,43] and lipase immobilization [44,45,46,47]. However, to the best of our knowledge, previous studies using PVA for enzyme immobilization have focused on organic systems with neutral reaction environments, while little attention has been paid to aqueous reaction systems with acidic or alkaline environments. In particular, GAD immobilization by PVA has not been widely studied. It is thus necessary to evaluate the feasibility of using PVA for GAD immobilization support to improve GABA isolation and biological production.

In this study, polyvinyl alcohol with sodium alginate was used to immobilize glutamate decarboxylase by forming PVA/SA GAD microspheres (PVA/SA–GADMs). The GAD was from *Lactobacillus brevis* CGMCC 1306 isolated in our lab, which was a homodimer [48] The effects of the PVA concentration, SA concentration, PVA: SA ratio, and PVA–SA: GAD ratio on spheronization and enzyme activity were assessed. The preparation conditions were optimized and the mechanical strength and external and inner morphology of the PVA/SA–GADMs were described. Then, PVA/SA–GADMs enzyme activity under a series of different pH and temperature conditions and new apparent *K*_m_ were studied to evaluate the improvements of immobilization on the catalytic and stability characteristics of GAD. Furthermore, to demonstrate the feasibility and robustness of the PVA/SA–GADMs for continuous GABA production, batch recycling catalysis stability and fixed-bed column catalyst operation stability were, respectively, designed and tested. This study aims to investigate a new GAD immobilization method using PVA–SA entrapment, to explore the effects of immobilization on GAD enzyme properties. Ultimately, the goal is to map out an entire flow scheme strategy covering the “PVA/SA–GADMs preparation-property characterization-fixed bed column catalyst operation” to enable the continuous scale-up of GABA green biological production.

## 2. Results and Discussion

### 2.1. GAD Immobilization

#### 2.1.1. Effects of Synthesis Conditions on the PVA/SA–GADMs Structure

In this study, the immobilization of glutamate decarboxylase involved the use of PVA and saturated boric acid. Sodium alginate was added to enhance the properties of microspheres. One purpose of the immobilization technique was to get good catalysis performance from the PVA/SA–GADMs. The catalysis characteristics for the immobilization of GAD were significantly influenced by the microsphere structure. So, the effects of the three synthesis conditions were consequently explored as follows: spheronization, gel diameter, and the effect of synthesis conditions on the mechanical strength of the PVA/SA–GADMs structure. To understand how the PVA and SA concentrations influence microsphere spheronization, gel diameter, and mechanical strength specifically, 6–10% (*w*/*v*) PVA and 1–3% (*w*/*v*) SA concentrations were selected for testing and the results are shown in Table 1, Table 2 and Table 3.

The results show that the 8–10% PVA concentration is appropriate for microsphere preparation, as lower PVA concentrations act as surfactants, which generate bubbles and disturb microsphere formation, while higher PVA concentrations have a high viscosity and are thus difficult to use in dripping operations. PVA concentration increases also lead to increases in the microsphere diameter and mechanical strength. The mechanical strength of the microspheres increased significantly with the 10% PVA concentration, and this was also found to be the optimal enzyme catalyst activity condition for PVA/SA–GADMs. Furthermore, as the SA concentration increased, the PVA/SA–GADMs diameter decreased and the mechanical strength slightly increased, and thus the 1–2% SA concentration could sufficiently meet the needs and was used in the subsequent investigations.

#### 2.1.2. Characterization of the PVA/SA GAD Microspheres

**Figure 1:**
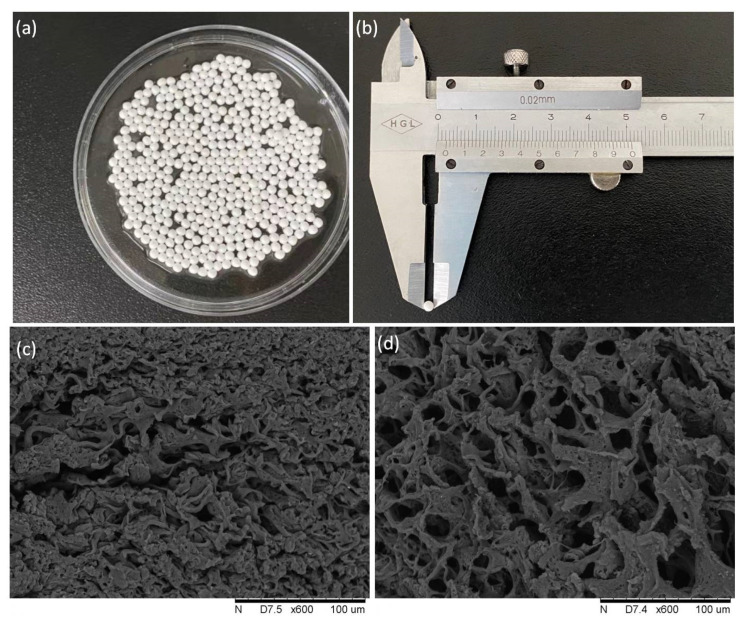
Morphological characteristics of PVA/SA–GADMs (**a**) PVA/SA–GADMs appearance (**b**) PVA/SA–GADMs diameter (**c**,**d**) PVA/SA–GAMs SEM photographs near the external area and internal area.

PVA/SA–GADMs are white, spherical, or ellipsoid in shape, with smooth surfaces, and diameters ranging from 2–3 mm. (Figure 1a,b). The SEM images showed that the areas near the external surface and inner structure of the PVA/SA–GADMs were smooth and ordered (Figure 1c,d). The pores were symmetrical distributed over the area near the surface and internal space, and the structure enabled the PVA/SA–GADMs to proceed mass transfer via the abundant transfer space [49]. At the same time, the pore distribution in the internal regions of microspheres was looser than that at the surface area, and this could be the result of the dramatic interactions between the SA and CaCl_2_ in the PVA/SA–GAD mixture when being dropped into the boric acid–CaCl_2_ solution. SA and CaCl_2_ cross linked rapidly and formed a mechanically strong and robust polyporous “membrane” which could protect the reaction between the PVA and boric acid gradually to form a loose internal structure. The structure could resist mechanical shearing during the PVA/SA–GADMs catalytic process and reduce the mass transfer resistance inside the microsphere, as the loose inner crosslinks were more readily available for enzyme–substrate binding. The expansion rate of the PVA/SA–GADMs was determined to be 550%. The high water-holding capacity makes the PVA/SA capsule a suitable tool for water-soluble enzyme immobilization.

#### 2.1.3. Effects of Synthesis Conditions on the Properties of the PVA/SA–GADMs Catalysis

**Figure 2:**
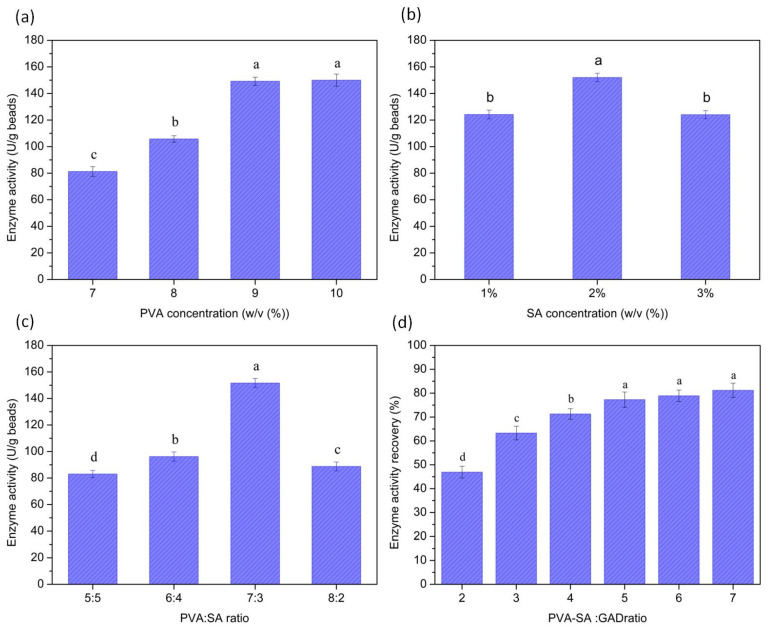
Effects of the synthesis conditions on the properties of the PVA/SA–GADMs catalyst. Effects of the (**a**) PVA concentration, (**b**) SA concentration, (**c**) PVA: SA ratio on PVA/SA–GADMs catalyst activity, and (**d**) PVA–SA: GAD ratio on PVA/SA–GADMs enzyme activity recovery. Data are expressed as the mean ± SD. Different lower case above the error bar indicate significant differences, where *p* < 0.05 indicates a significant difference (ANOVA, α = 0.05, Waller–Duncan test).

While synthesis conditions affect the microspheres’ structure, they simultaneously influence the properties of the PVA/SA–GADMs catalysis. The performance of the PVA/SA–GADMs catalysis with different PVA and SA concentrations, as well as PVA: SA and PVA–SA: GAD ratio is shown in Figure 2. As expected, the above factors significantly affected the properties of the PVA/SA–GADMs catalysis (*p* < 0.05). Changes in PVA concentration, SA concentration, and the PVA: SA ratio led to changes in enzyme activity. Moreover, the enzyme activity recovery was significantly different between different PVA–SA: GAD ratio groups. Enzyme activity per unit PVA/SA–GADMs mass tends to be stable until the PVA concentration reaches 9–10% (Figure 2a). While the 2% SA and 7:3 PVA/SA ratio gained the highest enzyme activity per unit PVA/SA–GADMs mass (Figure 2b,c). SA is composed of L-mannuronic acid and b-D-guluronic acid via a 1,4-glucosidic bond, which is abundant in hydroxyl. There is considered to be a delicate balance between the PVA and SA content within the PVA/SA–GADMs, and this influences the PVA/SA–GADMs catalyst performance. A small increase in the SA concentration will subsequently increase the opportunities for hydroxyl to bind with boron atoms which increases the strength and compaction of SA–boric acid–PVA. On the other hand, large steric hindrance between the SA side chains also leads to an increase in mass transfer resistance and a decrease in enzyme activity. Consequently, there was an optimum peak value for the SA concentration to PVA/SA–GADMs performance. A similar trend was found for the PVA: SA ratio. When the ratio was below or above the optimal 7:3 ratio, the enzyme activity decreased sharply due to bad spheronization.

The enzyme active recovery increased with the PVA/SA: GAD ratio and achieved a stable phase at a 5:1 ratio (Figure 2d). The results reflect the relationship between enzyme loading and the PVA–SA: GAD ratio. In the initial ratio, the PVA/SA mixture was superfluous which ensured that GAD was almost entirely entrapped, and consequently, as the PVA–SA ratio increased, so did the active recovery of the immobilized enzyme. When the ratio exceeded 5:1, extra PVA/SA could not load the enzyme, and the total enzyme active recovery tended to remain stable. The “empty” PVA/SA–GADMs mixed with “loaded” PVA/SA–GADMs led to enzyme activity decreases per unit microsphere mass. Consequently, the 5:1 ratio was determined to be optimal and used in subsequent investigations.

### 2.2. PVA/SA–GADMs Enzyme Properties and Applications

#### 2.2.1. Effects of pH on the PVA/SA–GADMs Enzyme Activity and Stability

The application of PVA/SA–GADMs is determined by catalysis performance under different conditions. Because GAD catalysis is an H^+^ consuming process, the pH gradually increases in the free GAD catalytic system. The reaction rate reduces because of the condition of being outside of the enzyme optimum pH. To solve this problem, it is necessary to study the effects of pH on PVA/SA–GADMs and improve the catalysis performance, expanding the application scope of immobilized GAD.

**Figure 3:**
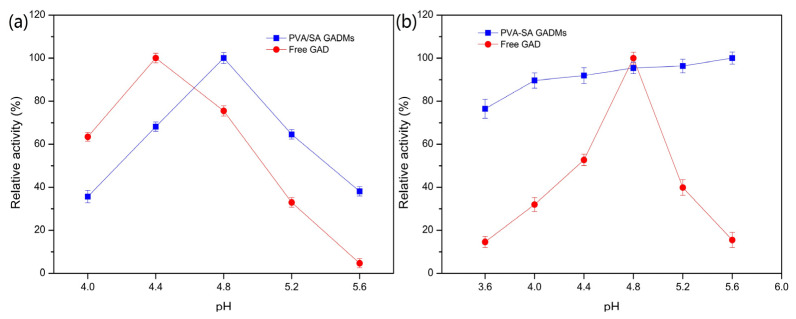
Effects of pH on the enzyme activity and stability of free GAD and PVA/SA–GADMs. Effects of pH on the (**a**) enzyme activity and (**b**) enzyme stability of free GAD and PVA/SA–GADMs.

To determine the optimum pH for immobilized GAD, PVA/SA–GADMs enzyme activity was tested in pH conditions ranging from 4.0–5.6. Free GAD relative activity was also tested as a reference (Figure 3a). The data shows that the optimum pH for the immobilized GAD was 4.8, which differed to the optimum pH for free GAD, which was 4.4, and the relative activity of the PVA/SA–GADMs was better than that of the free GAD when there was a pH > 4.8. This was because the PVA–SA gel beads contained high quantities of hydroxyl and the electronegativity of the oxygen atom in the hydroxyl could attract local H^+^ which then lowered the pH around the gel microenvironment. The low pH microenvironment could buffer the influence of the pH increase in the bulk liquid and the immobilized GAD could thus appear to have a higher optimum pH when compared with free GAD [50].

The pH stabilities of the PVA/SA–GADMs and free GAD after incubation in pH conditions ranging from 3.6–5.6, are shown in Figure 3b. The PVA/SA–GADMs showed a strong level of robustness in pH conditions ranging from 4.0–5.6, as approximately 90% of the enzyme activity was maintained, when compared with the free GAD enzyme activity which decreased sharply to 40% in pH conditions outside of 4.8. This was because the PVA–SA crosslink strength increased gradually as the pH increased, and the pH around the microenvironment was lower than that in the bulk liquid due to the attraction between the oxygen atom of PVA and the H^+^. Both ensured immobilized GAD was more tolerant to higher pH conditions and the performance showed improved stability in a wider range of pH conditions. As GAD catalysis is H^+^ consuming, and the pH of bulk liquids increases gradually, GAD could be rapidly denatured, thus extending the range of pH stability towards the alkaline direction will ultimately have practical benefits for batch and fixed-bed column catalysis operations to enable the continuous production of GABA.

#### 2.2.2. Effects of Temperature on PVA/SA–GADMs Enzyme Activity and Stability

**Figure 4:**
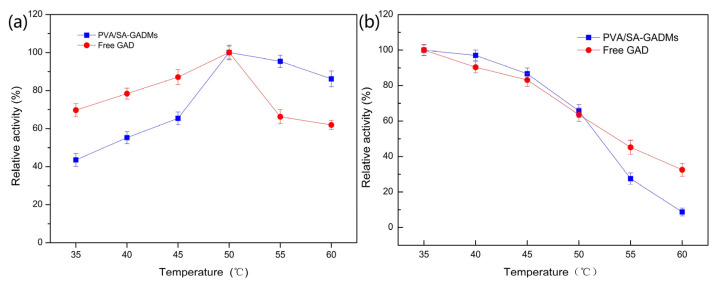
Effects of temperature on the enzyme activity and stability of free GAD and PVA/SA–GADMs. (**a**) Effects of temperature on the enzyme activity and (**b**) Thermal stability of free GAD and PVA/SA–GADMs.

The temperature also affects the catalysis performance of immobilized GAD. To determine the optimum temperature for the immobilized GAD, PVA/SA–GADMs activity was tested in different temperature conditions, ranging from 35–60 °C and free GAD was used as a reference (Figure 4a). The thermal stability results are shown in Figure 4b.

The optimum catalysis temperature of 50 °C for PVA/SA–GADMs with free GAD is shown in Figure 4a. The relative activity of the PVA/SA–GADMs showed a similar trend with the free GAD below 50 °C. However, the PVA/SA–GADMs maintained 95.3% and 86.1% activity levels, respectively, while the activity levels for the free GAD were 66.2% and 61.9%, respectively, after 10 min of the catalytic reaction at 55 °C and 60 °C; this implies that the immobilized GAD has better catalyst capabilities in high temperature conditions. The reason for this is that the heat plays a two-sided role in enzyme catalytic reactions, by boosting the reaction rate with the energy input, and inactivating enzymes by destroying the enzyme spatial structure. The former dominates the reaction under 50 °C in both immobilized GAD and free GAD, which led to a similar trend. The latter dominated at >50 °C, and this caused a sharp decrease in the enzyme activity of the free GAD. However, the net structure of the PVA–SA gels absorbed the extra heat from the bulk liquid, which limited the free stretching of the GAD, protected the GAD structure breakdown, and resulted in the immobilized GAD having improved catalyst capabilities [51].

The PVA/SA–GADMs thermal stability is better than that of the free GAD < 55 °C, as the PVA/SA–GADMs enhanced the stability of the enzyme by decreasing the speed of enzyme inactivation (Figure 4b). At temperatures >55 °C, there was a decrease in PVA/SA–GADMs activity when compared with the free GAD. This phenomenon could be the result of PVA/SA–GADMs structures being mechanically unstable and the start of a melt [52,53]. The leakage of GAD to the bulk liquid weakened the capabilities of the PVA/SA–GADMs catalyst, and consequently, the PVA/SA–GADMs showed a different performance trend with the long-term heat treatment. Although 55 °C is a turning point for PVA/SA–GADMs thermal stability, there were no large effects on the PVA/SA–GADMs application. The PVA/SA–GADMs showed robust thermal stability at 35–40 °C and this may thus be the optimum temperature range for long-term GABA production, when considering the economic benefit from the energy cost.

#### 2.2.3. Kinetic Study of the PVA/SA–GADMs

**Figure 5:**
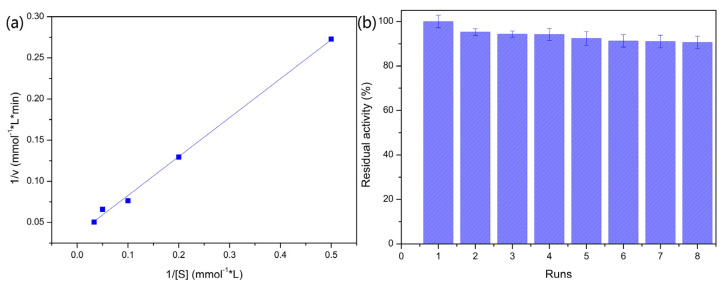
Michaelis–Menten plot and batch operation stability for the PVA/SA–GADMs (**a**) Michaelis–Menten plot for the PVA/SA–GADMs and (**b**) Batch operation stability for the PVA/SA–GADMs.

A Michaelis–Menten plot for the PVA/SA–GADMs is shown in Figure 5a. The apparent K_m_ for the immobilized GAD was 13.3 mmol/L, while that of the free GAD was 30.9 mmol/L. This indicates that the PVA–SA entrapped GAD could increase the enzymes apparent affinity for the substrate. The potential interactions of the PVA–SA side chain with immobilized GAD may lead to a slight change in enzyme structure which makes it easier for the catalytic active site to bind substrates. Furthermore, the interactions between the electronegative carboxyl group of the L-sodium glutamate substrate and the boron in PVA–SA gel beads could lead to an increase in the substrate concentration around the microenvironment, which showed a higher apparent affinity for the substrate. Consequently, PVA/SA entrapment is an ideal method by which to achieve GAD immobilization. 

#### 2.2.4. Operation Stability for the PVA/SA–GADMs Recycling Batch

The PVA/SA–GADMs were found to have good performance with repeated use, as there was up to 90.6% enzyme activity retention after eight cycles (Figure 5b). This shows that the PVA/SA–GADMs have good mechanical strength and operation stability. PVA/SA entrapment is thus suitable for GABA batch production methods.

#### 2.2.5. Continuous Fixed-Bed Column Catalyst Operation for PVA/SA–GADMs

**Figure 6:**
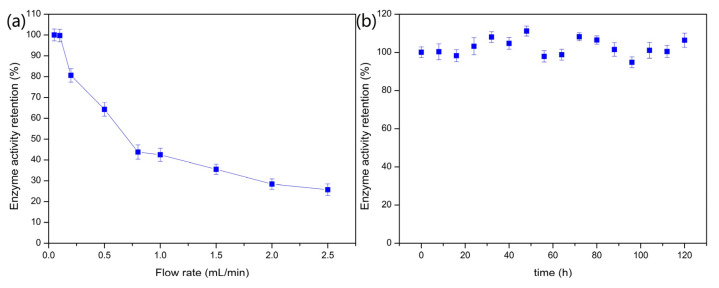
Effects of substrate flow rate on enzyme activity retention and catalysis stability of fixed-bed column operation. (**a**) Effects of substrate flow rate on enzyme activity retention in a fixed-bed column operation and (**b**) Fixed-bed column catalyst operation stability.

To further explore the continuous GABA production capabilities of PVA/SA–GADMs, the microspheres were prepared and packed in a fixed-bed column system with enough catalyst to last for 120 h. The effects of the substrate flow rate on the GABA conversion rate in the fixed-bed column are shown in Figure 6a. The immobilized GAD enzyme activity retention was close to 100% with a substrate flow rate of 0.05 mL/min and 0.1 mL/min and decreased as the substrate flow rate increased. The immobilized GAD enzyme activity retention was maintained at a level close to 100% with the 0.1 mL/min substrate flow rate after 120 h of continuous catalyst operation (Figure 6b). The performance of the fixed-bed column catalyst highlights the mechanical strength, high tolerance to alkaline pH, and good thermostability of the PVA/SA–GADMs that has been previously described.

Overall, the PVA/SA immobilization method broadened the enzyme stability scope and improved the apparent affinity for the substrate, and that PVA/SA–GADMs could be repeatedly used as a batch and fixed-bed column catalyst. Therefore, PVA/SA entrapment is a promising strategy for continuous large-scale GABA production. The results also showed that PVA/SA entrapment is suitable for aqueous reaction systems with acidic environments, which filled some of the gaps for PVA/SA application in enzyme immobilization. Based on these results, PVA/SA entrapment is a potential immobilization method for other water-soluble enzymes and could be designed as a universal immobilization tool suitable for continuous, large-scale active-substance production. In future research, the integration of new materials into PVA/SA patterns can be considered to strengthen their crosslinks so as to improving the thermostability of microspheres in high temperatures. Consequently, the applicability of this method could be increased to a broader active-substance scope.

## 3. Materials and Methods

### 3.1. Materials

Polyvinyl alcohol (DP: 1750 ± 500), sodium alginate, boric acid, calcium chloride (CaCl2), sodium acetate, acetic acid, and tetrahydrofuran were purchased from Sinopharm Chemical Reagent Co., Ltd. (Shanghai, China). While the tryptone, yeast powder, and L-sodium glutamate (L-MSG) were purchased from Sangon Biotech Co., Ltd. (Shanghai, China) and the chromatographic grade methanol was purchased from Oceanpak Alexative Chemical., Ltd. (Goteborg, Sweden). GABA producing *Lactobacillus brevis* CGMCC 1306 was isolated in our laboratory from fresh milk.

### 3.2. Methods

#### 3.2.1. GAD Immobilization and Optimization

PVA (5–12%, *w*/*v*) was premixed with SA (1–3%, *w*/*v*) at different ratios (80/20–50/50, *w*/*w*) and tenderly blended. The GAD (0.35 mg/mL) was mixed with PVA–SA solution at different ratios (2/1–7/1, *v*/*v*). The PVA–SA–GAD solution was then extruded from a 0.5 mm diameter single nozzle at 0.5 mL/min and dripped into the saturated boric acid and calcium chloride (1%, *v*/*v*) solution with stirring at 350 rpm. The PVA/SA–GADMs formed were stored at 4 °C for 8 h to allow them to harden in the solution. The gel beads were then filtered and washed using deionized water.

#### 3.2.2. Characterization of PVA/SA–GADMs

The morphological characteristics of PVA/SA–GADMs were observed using scanning electron microscopy (SEM, Hitachi-TM3000). The sample was cut using a surgical knife and then glued to the sample table using conductive glue. The pretreated sample was then sprayed with a layer of gold and images obtained using SEM.

Mechanical strength was measured using uniaxial compression tests. First, 50 PVA/SA–GADMs were randomly selected, the gels located on the tray of the electronic scales were then pressed vertically until fracture, and the data was recorded and used to calculate the average fracture initiation pressure value as the mechanical strength.

The diameter was measured using vernier calipers. First, 50 PVA/SA–GADMs were randomly selected, and the average value of the measurements was recorded as the diameter.

The expansion rate was evaluated using the gel weight changes before and after the drying process using Equation (1), which was as follows:

*S = (W − W*_0_*)/W*_0_ (1)

where *S* is the expansion rate, *W* is the PVA/SA–GADMs weight after incubating in distilled water for 24 h under 25 °C, and *W*_0_ is the PVA/SA–GADMs constant weight after wet gel drying in the vacuum oven.

#### 3.2.3. Enzyme Activity Assays

Enzyme activity assays were in accordance with the description by Ueno et al. [54]. First, 500 μL GAD or 1 g PVA/SA–GADMs was added to 5 mL L-sodium glutamate solution (final concentration 10 mM with 0.01 mM phosphopyridoxal in acetate buffer, pH 4.4) and incubated for 10 min at 37 °C, and the reaction was ended by placement in a boiling water bath for 3 min or filtration. The GABA concentration was measured using high performance liquid chromatography (HPLC) (SHIMADZU, LC-2030). The sample for the HPLC test was mixed with 0.5 mol/L NaHCO_3_ and 8 g/L dansyl chloride–acetone solution, and it was reacted for 1 h at 30 °C in the dark. The mixture was filtered through a 0.22-μm microfiltration membrane and tested with a chromatographic column (Elite, Hypersil ODS2 C18) using a gradient elution procedure of mobile phases A and B. Mobile phase A was methanol, and mobile phase B was a complex of tetrahydrofuran, methanol, and 0.05 mol/L sodium acetate (5:75:420, *v/v/v*) [55,56]. One international unit (IU) of GAD activity was defined as the enzyme quantity required for 1 µmol GABA production per min under optimum assay conditions. 

#### 3.2.4. Enzymatic Properties and Kinetic Parameter Measurements

PVA (10%, *w*/*v*) was premixed with SA (2%, *w*/*v*) at a 7:3 ratio, and the mixture was then mixed with GAD at a 5:1 ratio to prepare the PVA/SA–GADMs. The enzymes optimum pH was tested as the free GAD and PVA/SA–GADMs were added into L-sodium glutamate solutions with pH values ranging from 3.6–5.6, and the relative activity in the different pH conditions evaluated. The relative activity was defined as the ratio of tested activity to the maximum enzyme activity, which is recorded at the optimum pH as 100%. The optimum temperature for the enzymes was assessed by evaluating the effects of temperatures ranging from 35–60 °C on the catalytic process. The pH stability was tested 1 h after incubation under pH conditions ranging from 3.6–5.6, prior to the catalytic reaction. Thermostability was tested after 1 h of incubation in temperatures ranging from 35–60 °C. The *K*_m_ was then calculated using the Michaelis–Menten equation under 2, 5, 10, 20 and 30 mM for the initial L-sodium glutamate concentration.

#### 3.2.5. PVA/SA–GADMs Batch Recycling Operation

To analyze the batch recycling operation, 1 g PVA/SA–GADMs were added to 5 mL 10 mM L-sodium glutamate solution and reacted at a pH of 4.8 and 37 °C for 10 min. The PVA/SA–GADMs were then collected by filtering the reaction liquid and washed 2–3 times with acetate buffer (pH 4.8). Then, 1 mL of filtrate was taken for the enzyme activity assay, and PVA/SA–GADMs were used again for the catalysis reaction. This process was then repeated 8 times.

#### 3.2.6. PVA/SA–GADMs Continuous Fixed-Bed Column Catalyst Operation

**Figure 7:**
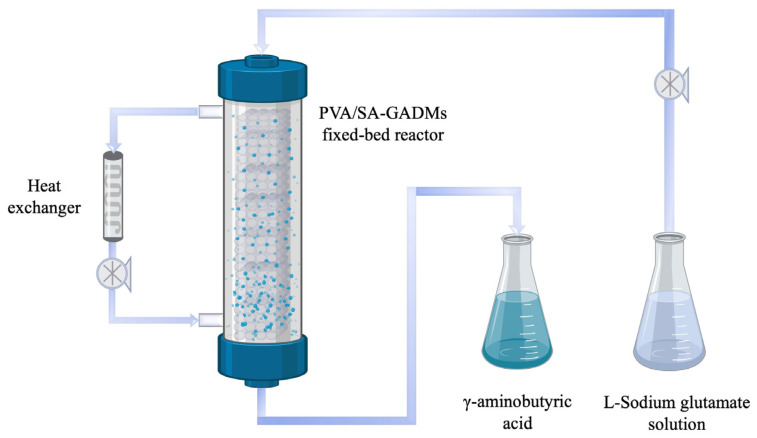
Continuous fixed-bed column catalyst operation flow diagram. The L-sodium glutamate solution is transported into a polyvinyl alcohol/sodium alginate-glutamate decarboxylase microspheres (PVA/SA–GADMs) fixed-bed reactor by a pump, and it is converted into γ-aminobutyric acid by immobilized GAD within the reactor. Then, γ-aminobutyric acid is collected. The heat exchanger is used to maintain a constant temperature for the fixed-bed reactor.

A 5 mL plastic chromatographic column (sinopae, DE02-FAST-E) was selected as the fixed-bed column reactor and PVA/SA–GADMs were packed into the column. A 10 mM L-sodium glutamate solution (pH 4.8) was passed through the down-flow column reactor at 37 °C under different flow rates (0.05, 0.1, 0.2, 0.5, 0.8, 1.0, 2.0, and 2.5 mL/min) using a digital syringe pump (longer pump, LSP01-1A). The effluent was collected from the bottom of the column and used for enzyme activity retention analysis by HPLC, to determine the optimum flow rate. The process was then repeated using a 0.1 mL/min L-sodium glutamate solution flow rate and the effluent collected at 8 h intervals for fixed-bed column catalyst operation robustness analysis (Figure 7).

#### 3.2.7. Statistical Analysis

Experiments were conducted in triplicate and all data are displayed as the means ± SD. Data analysis including the determination of statistical significance was conducted using Origin 8.5 and IBM SPSS Statistics 26. The Waller–Duncan test was used to analyze statistical significance variability between samples using ANOVA with a significance level of 95% (α = 0.05).

## 4. Conclusions

In this study, GAD was successfully immobilized using a polyvinyl alcohol–sodium alginate cross-linked network of microspheres. The optimum preparation conditions for the PVA/SA–GADMs were determined to be 10% (*w*/*v*) PVA with a 7:3 ratio for the 2% (*w*/*v*) SA, and a 5:1 ratio for the PVA–SA mixture with GAD, as this resulted in good mechanical strength and catalytic performance. PVA–SA entrapment improved the thermostability at <50 °C, and notably expanded the pH range that enabled catalysis stability. PVA/SA–GADMs showed approximately 90% activity between the pH range of 4.0–5.6. The affinity to the substrate with immobilized enzyme was also improved when compared with free GAD that had a *K*_m_ of 13.3 mmol/L. PVA/SA–GADMs show good batch operation stability, which maintained an activity level of >90.6% after eight catalytic cycles. To the best of our knowledge, this is the first study to use the PVA–SA entrapment strategy for the immobilization of GAD. In addition, this work is the first to confirm the feasibility of immobilized GAD microspheres in a continuous fixed-bed column catalyst operating system over 120 h with a near-100% enzyme activity retention. The strategy is efficient, environmentally friendly, and low cost. The results thus provide a solid foundation and a promising applied technical orientation for large scale green biological GABA manufacturing in the future.

## Figures and Tables

**Table 1:** Spheronization of the PVA/SA–GADMs * with different PVA concentrations.

PVA Concentration (*w*/*v*)	Spheronization
<6%	Microsphere not formed
6–8%	Bad spheronization with tail
8–10%	Good spheronization
>10%	High viscosity hard for dripping operation

* PVA/SA–GADMs is the abbreviation of polyvinyl alcohol/sodium alginate-glutamate decarboxylase microspheres.

**Table 2:** Diameters of the PVA/SA–GADMs with different PVA/SA concentrations.

PVA Concentration (*w*/*v*)	SA Concentration (*w*/*v*)	Diameter (mm)
6%	1%	1.85 ± 0.05
	2%	1.78 ± 0.08
	3%	1.76 ± 0.05
8%	1%	1.90 ± 0.02
	2%	1.78 ± 0.05
	3%	1.75 ± 0.05
10%	1%	2.12 ± 0.05
	2%	2.08 ± 0.05
	3%	1.95 ± 0.02

**Table 3:** Mechanical strength of PVA/SA–GADMs with different PVA/SA concentrations.

PVA Concentration (*w*/*v*)	SA Concentration (*w*/*v*)	Mechanical Strength (N)
6%	1%	1.35 ± 0.2
	2%	1.38 ± 0.2
	3%	1.42 ± 0.2
8%	1%	3.01 ± 0.2
	2%	3.08 ± 0.5
	3%	3.32 ± 0.2
10%	1%	5.40 ± 0.5
	2%	5.42 ± 0.2
	3%	5.48 ± 0.2

## Data Availability

Data sharing not applicable.
